# Structural Studies of Fucosylated *N*-Glycans by Ion Mobility Mass Spectrometry and Collision-Induced Fragmentation of Negative Ions

**DOI:** 10.1007/s13361-018-1950-x

**Published:** 2018-05-22

**Authors:** David J. Harvey, Weston B. Struwe

**Affiliations:** 1 Glycobiology Institute, Department of Biochemistry, University of Oxford, South Parks Road, Oxford, OX1 3QU UK; 2 Present Address: Target Discovery Institute, Nuffield Department of Medicine, University of Oxford, Roosevelt Drive, Oxford, OX3 7FZ UK; 3 Chemistry Research Laboratory, Department of Chemistry, University of Oxford, 12 Mansfield Road, Oxford, OX1 3TA UK

**Keywords:** *N*-Glycans, Fucosylation, Negative ion, Ion mobility, Fragmentation

## Abstract

There is considerable potential for the use of ion mobility mass spectrometry in structural glycobiology due in large part to the gas-phase separation attributes not typically observed by orthogonal methods. Here, we evaluate the capability of traveling wave ion mobility combined with negative ion collision-induced dissociation to provide structural information on *N*-linked glycans containing multiple fucose residues forming the Lewis^x^ and Lewis^y^ epitopes. These epitopes are involved in processes such as cell-cell recognition and are important as cancer biomarkers. Specific information that could be obtained from the intact *N*-glycans by negative ion CID included the general topology of the glycan such as the presence or absence of a bisecting GlcNAc residue and the branching pattern of the triantennary glycans. Information on the location of the fucose residues was also readily obtainable from ions specific to each antenna. Some isobaric fragment ions produced prior to ion mobility could subsequently be separated and, in some cases, provided additional valuable structural information that was missing from the CID spectra alone.

**Graphical abstract:**
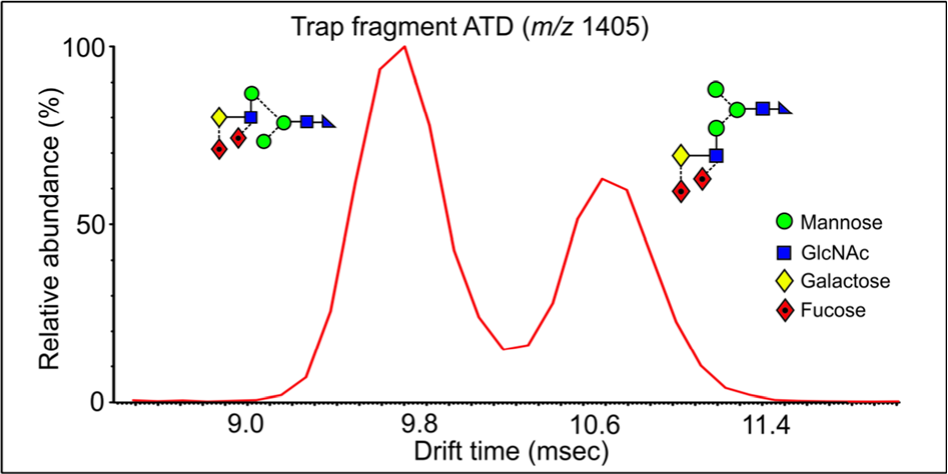
ᅟ

ᅟ

## Introduction

Approximately half of all proteins are estimated to be glycosylated either at asparagine in an Asn-Xxx-Ser(Thr) motif where Xxx is any amino acid except proline (termed *N*-linked) or to serine or threonine (termed *O*-linked). These glycans are involved in many processes, such as protein folding, cell-cell recognition, and protein turnover [1], and methods for their structural determination have been developed by many research groups, e.g., [2–8]. Typically, *N*-glycans are released from the glycoproteins by chemical or enzymatic processes and analyzed by techniques such as high-performance liquid chromatography (HPLC), often combined with exoglycosidase digestion or mass spectrometry (MS) [9–13]. Mass spectrometry is most commonly performed in positive ion mode which, although useful in assigning compositions and sequence information, is not so useful for determination of glycan topology without the use of additional techniques such as permethylation. Negative ion collision-induced dissociation (CID) spectra, on the other hand, have been shown to provide more extensive structural information directly from native glycans [14–21] and have the advantage over positive ion CID that isomers are usually easily identified in mixed spectra by mass-different cross-ring fragment ions. One of the problems encountered with structural analyses of *N*-glycans is that many of them are typically released as mixtures of isomers and often in very small quantities [22–24] that are frequently contaminated with other endogenous and extraneous material. Negative ion CID alleviates the isomer problem particularly when accompanied by ion mobility and this latter technique has also proved to be invaluable for isolating glycan-derived ions from contaminated mixtures, thus minimizing extensive pre-mass spectrometric clean-up strategies with their accompanying sample losses [25, 26]. Thus, ion mobility combined with negative ion CID provides an excellent method for *N*-glycan analysis [27–35].

We have developed these techniques for the analysis of high-mannose [14, 15, 17, 36], hybrid [16, 17, 37], and complex *N*-glycans [16, 17, 19, 20, 25, 37–41] but little attention has so far been paid to glycans with multiple fucose residues present on the core GlcNAc and on their antennae. Fucosylated *N*-glycans are encountered in many biological systems [42–49] where they are involved in processes such as cell-cell recognition, embryo development, and disease processes [50, 51]. In particular, we were interested in fucosylated *N*-glycans containing the Lewis^x^ (Gal-1→4(Fuc-1→3)GlcNAc) and Lewis^y^ ((Fuc-1→2)Gal-1→4(Fuc-1→3)GlcNAc) epitopes which can be upregulated in cancer [52–54] and which can be used as biomarkers [55]. This paper examines the use of negative ion CID for determining the structures of these compounds and extends earlier work by evaluating the use of ion mobility for isomer detection using both the native glycans and their collision-induced fragments. In particular, we were interested to see if ion mobility could provide structural information that was not present in the CID spectra. For example, work by Gray et al. [56] in positive ion mode shows that cross sections of some fragment ions contain information on glycan anomericity and it was of interest to see if similar information could be obtained from these negative ion spectra. Lewis^x^ and Lewis^y^-containing *N*-glycans used in this work were conveniently obtained from a sample of glycans that had been released from glycoproteins present in human parotid glands, and which have previously been shown [42, 43, 57, 58] to contain hybrid and complex bi-, tri-, and tetra-antennary *N*-glycans with multiple fucose residues decorating the antennae resulting in many of the glycans carrying the Lewis^x^ and Lewis^y^ blood group antigens. In our earlier study [43], glycan structures were mainly determined by the classical techniques of HPLC and exoglycosidase digestions, and it was of interest to compare the results of this study with information that could be obtained more easily with the modern techniques used here.

## Materials and Methods

### Materials

Methanol was obtained from BDH Ltd. (Poole, UK) and ammonium phosphate was from Aldrich Chemical Co. Ltd. (Poole). Dextran from *Leuconostoc mesenteroides* was obtained from Fluka (Poole, UK).

### Fucosylated *N*-Glycans

Most of the fucosylated glycans discussed in this paper were obtained from human parotid glands as described earlier [43]. The *N*-glycans were released from the glycoproteins by heating with hydrazine (40 mL) at 85 °C for 12 h. After removal of the excess of hydrazine, the glycans were re-*N*-acetylated with acetic anhydride in a saturated aqueous solution of sodium bicarbonate. Sodium salts were removed by Dowex AG 50W2X12 chromatography and peptides were removed by chromatography on microcrystalline cellulose in butanol/ethanol/water (4:1:1, by vol.) at room temperature. Samples were dried with a rotary evaporator and stored at − 18 °C. Other glycans were obtained from human IgA by in-gel peptide-*N*-glycosidase F (PNGase F)-release [59] as described earlier [36].

### Purification of Glycans for Mass Spectral Analysis

All glycan samples were cleaned with a Nafion® 117 membrane as described earlier by Börnsen et al. [60]. Samples were then dissolved in a solution of methanol/water (1:1, *v*/*v*) containing ammonium phosphate (0.05 M, to maximize formation of [M + H_2_PO_4_]^−^ ions, the ions usually encountered from biological samples) and centrifuged at 10,000 rpm (9503×*g*) for 1 min to sediment any particulate matter before examination by mass spectrometry.

### Mass Spectrometry

Mass spectrometry was performed with a traveling wave ion mobility Synapt G2Si Q-TOF instrument (Waters Corp., Manchester, UK) [61] fitted with a nano-electrospray (n-ESI) ion source. Samples were infused through gold-coated borosilicate nanospray capillaries prepared in-house [62]. The instrument was set up as follows: ESI capillary voltage, 0.8–1.0 kV; sample cone voltage, 150 V; ion source temperature, 80 °C; ion mobility gas, nitrogen, 80 mL/min; T-wave velocity, 450 m/s; T-wave peak height 40 V, trap voltage, 60 V. Collision-induced dissociation was performed both before and after mobility separation in the trap and transfer cells respectively with argon as the collision gas. The instrument was externally mass-calibrated with sodium iodide and the mobility cell was calibrated with (Glc)_2–13_ glycans present in dextran (from *Leuconostoc mesenteroides*). Data acquisition and processing were carried out using the Waters Driftscope (version 2.8) software and MassLynx™ (version 4.1). The scheme devised by Domon and Costello [63] was used to name the fragment ions with the following exception: the subscript R (for reducing terminal) is used when general reference is made to loss or fragmentation of a GlcNAc residue from the reducing terminus of the glycan in order to avoid confusion caused by the subscript number changing as the result of altered chain lengths. Interpretation of the spectra followed rules developed earlier in this laboratory [14–17].

### CCS Estimation

Absolute helium and nitrogen collision cross sections (CCSs) of singly charged dextran oligomers (Glc_3_–Glc_13_), measured previously using a modified Synapt high-definition (HDMS) [64] (Waters) quadrupole/IMS/oa-TOF MS instrument containing a RF-confined linear (not traveling wave) drift tube ion mobility instrument, were used to estimate glycan CCSs from traveling wave ion mobility (TWIMS) data. [65–67]. Sample introduction and instrument set-up was the same as above. Estimation was performed with the method described by Thalassinos et al. [68] and compared with previous measurements made using the drift tube instrument [69].

## Results and Discussion

The sample of parotid glycans used for this work was the same as that was used for the earlier paper [43]. It had been stored at − 18 °C for some 20 years, yet the mass spectral profile of glycans showed no significant difference from the earlier profile confirming that any decomposition was negligible. Fragmentation was performed in negative ion mode in both the trap (before IM) and transfer (after IM) regions of the instrument with spectral interpretation following the rules developed from earlier experiments [14–17]. Because CID preceded mobility when performed in the trap region, the fragment ions could subsequently be separated to provide a potential source of additional structural information to that generated in transfer region CID experiments.

### Hybrid Glycans

**Figure 1:**
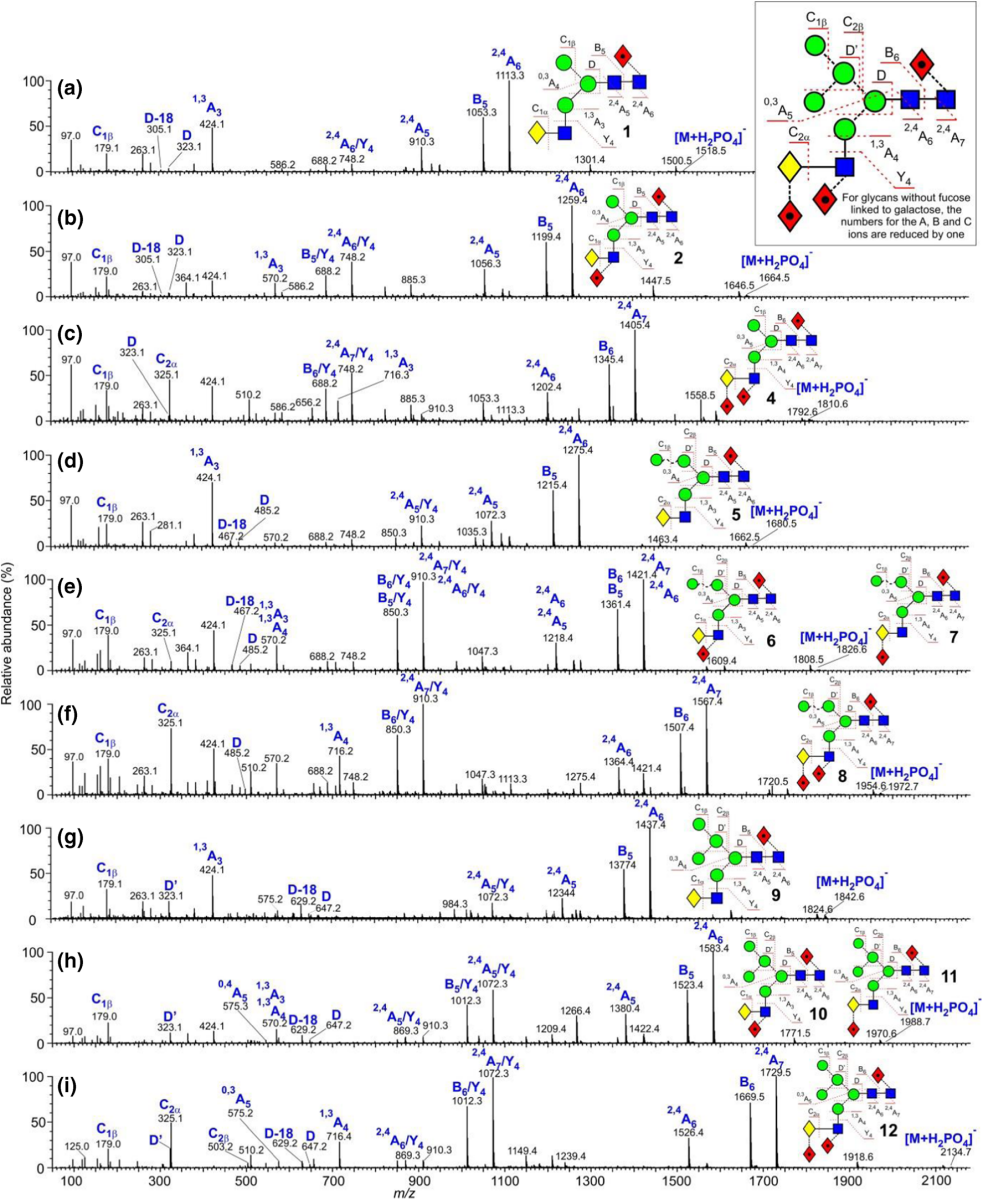
(**a**–**i**) Negative ion CID spectra (transfer region) of hybrid glycans obtained from human parotid glands. Symbols and linkages for the structural diagrams are as defined in the footnote of Table 1. Numbers in bold black are of the structures listed in Table 1. The inset to (**a**) and (**b**) shows an expanded representation of the fragmentation of the penta-fucosylated glycan **12**

Hybrid glycans with two, three, or four mannose residues in the 6-antenna and containing a 3-linked Gal-GlcNAc-antenna were present with one, two, and three fucose residues each (glycans **1**–**12**, Table 1, with glycan structures as described in Reference [70]). Their CID spectra, recorded in the transfer cell, are shown in Fig. 1. All the glycans contained one fucose residue attached to the 6-position of the reducing-terminal GlcNAc residue as shown by the masses of the ^2,4^A_R_. B_R-1_ and ^2,4^A_R-1_ ions (losses of 405, 465, and 608 mass units respectively). The B_R-1_ and ^2,4^A_R-1_ fragments also confirmed the structure of the β1-4-linked chitobiose and showed no further fucose substitution in the core GlcNAc residues.

**Table 1:**
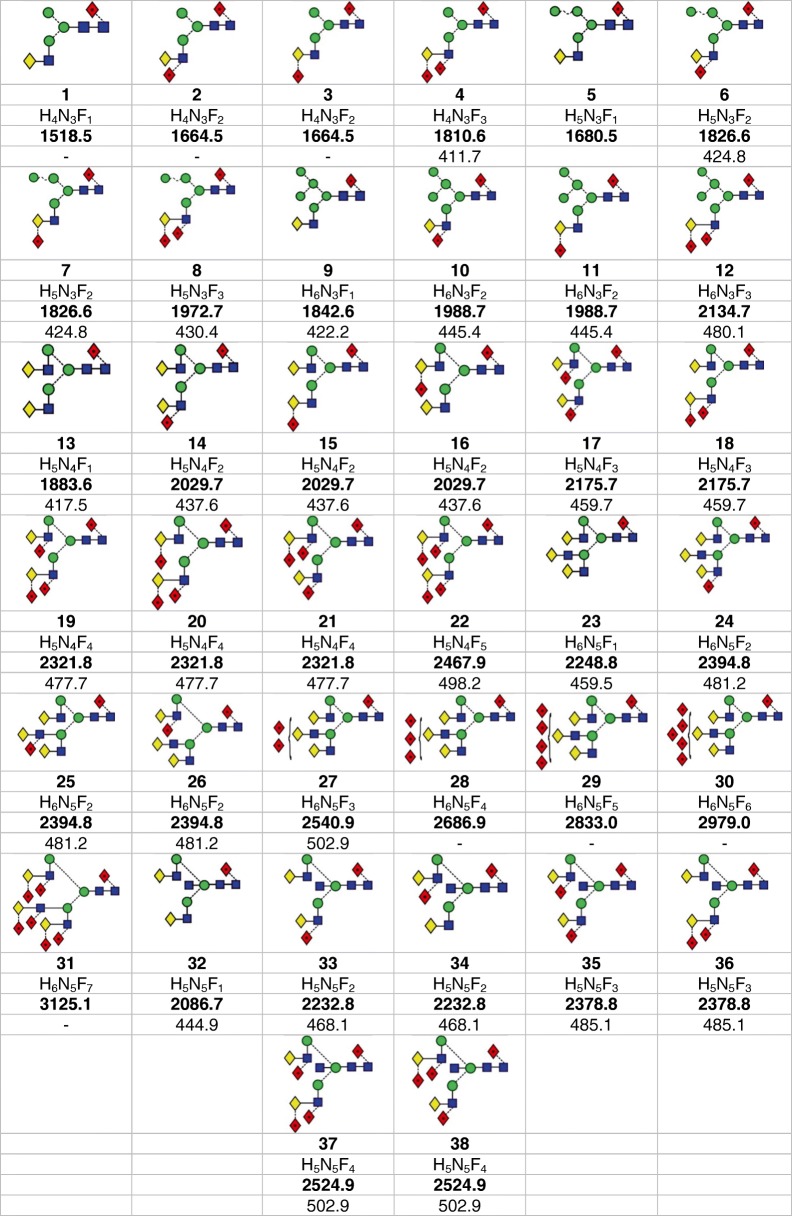
Structures, Masses (in Bold) and Nitrogen CCS Values (in Italics) for Human Parotid Fucosylated *N*-Glycans

Symbols used for the glycans are  = mannose,  = GlcNAc,  = galactose, and  = fucose. Solid lines connecting the symbols are β-linkages; broken lines are α-linkages. The angle of the lines shows the linkage position: | = 2-link, / = 3-link, - = 4-link, \ = 6-link. For more information, see [70]. The numbers, in **bold**, below the structures are the *m*/*z* value of the phosphate adducts and numbers in normal font are the collisional cross section (nitrogen) in Å^2^

The ion at *m*/*z* 424 in the spectrum of the mono-fucosylated glycan **1** is a ^1,3^A_3_ cross-ring fragment containing the Gal-GlcNAc chain. The earlier work on the parotid glycans showed that additional fucose residues were added to this chain in the 3-position of the GlcNAc or the 2-position of the galactose residues in this and all of the other glycans. The spectra of the di-fucosylated glycans, **2**, **6**, **7**, **10,** and **11** contained a prominent ion at *m*/*z* 570 corresponding to the ^1,3^A_3_ ion with an additional fucose residue. The virtual absence of a C_2_ ion at *m*/*z* 325 (Fuc-Gal) was consistent with most of the glycans at this mass containing the fucose 3-linked to the GlcNAc residue, as determined earlier by exoglycosidase digestion [43]. However, the spectra also contained an ion at *m*/*z* 424; this being the corresponding ion without fucose and, presumably, being the result of a secondary fragmentation involving loss of fucose. The presence of fucose on the GlcNAc residue of the 3-antenna produced a significant increase in the relative abundance of the ^2,4^A_R_/Y_4_ and B_R-1_/Y_4_ ions (*m*/*z* 748/688, 910/850, and 1072/1012 respectively).

The CID spectra of the tri-fucosylated glycans **4**, **8**, and **12** contained additional ^1,3^A_4_ ions at *m*/*z* 716 confirming the presence of two fucose residues on a single antenna. However, the spectra also contained corresponding ions at *m*/*z* 570 and 424 resulting from fucose neutral loss. The presence of the fucose residue linked to the galactose residue gave rise to a prominent C_2_ ion at *m*/*z* 325.

Estimated collisional cross sections (nitrogen) were measured against dextran oligomers and are listed in Table 1.

### Biantennary Glycans

#### Transfer Fragmentation

**Figure 2:**
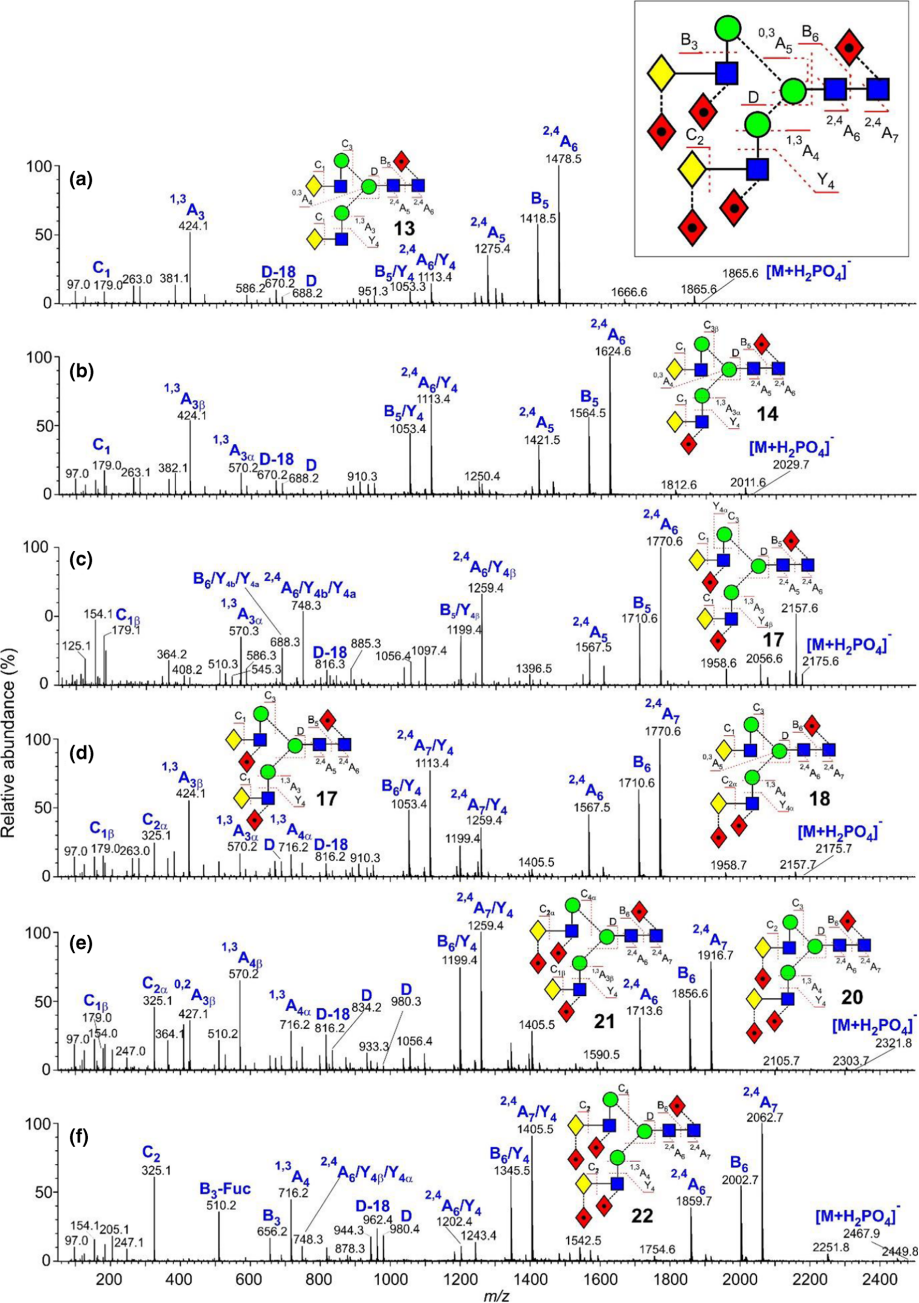
(**a**–**f**) Negative ion CID spectra (transfer region) of fucosylated biantennary complex glycans. The spectrum of the tri-fucosylated glycan **17** shown in panel (**c**) is from human IgA; the remainder of the spectra are from human parotid gland extracts. Symbols and linkages for the structural diagrams are as defined in the footnote to Table 1. The inset to panels (**a**) and (**b**) shows an expanded representation of the fragmentation of the tri-fucosylated glycan **22**

These glycans were present with one to five fucose residues and the CID spectra of those from the parotid glands, recorded earlier with a Waters Ultima Global Q-TOF instrument have been discussed briefly in an earlier publication [16]. These spectra were virtually identical with the transfer CID spectra recorded here with the Synapt G2Si instrument (Fig. 2). An additional biantennary glycan containing fucose on the core and two additional fucose residues on the GlcNAc residues of the antennae (glycan **17**) was found in the sample of released glycans from human IgA where the enzyme FUT2 was absent. This enzyme is responsible for adding a fucose residue to the 2-position of galactose.

**Figure 3:**
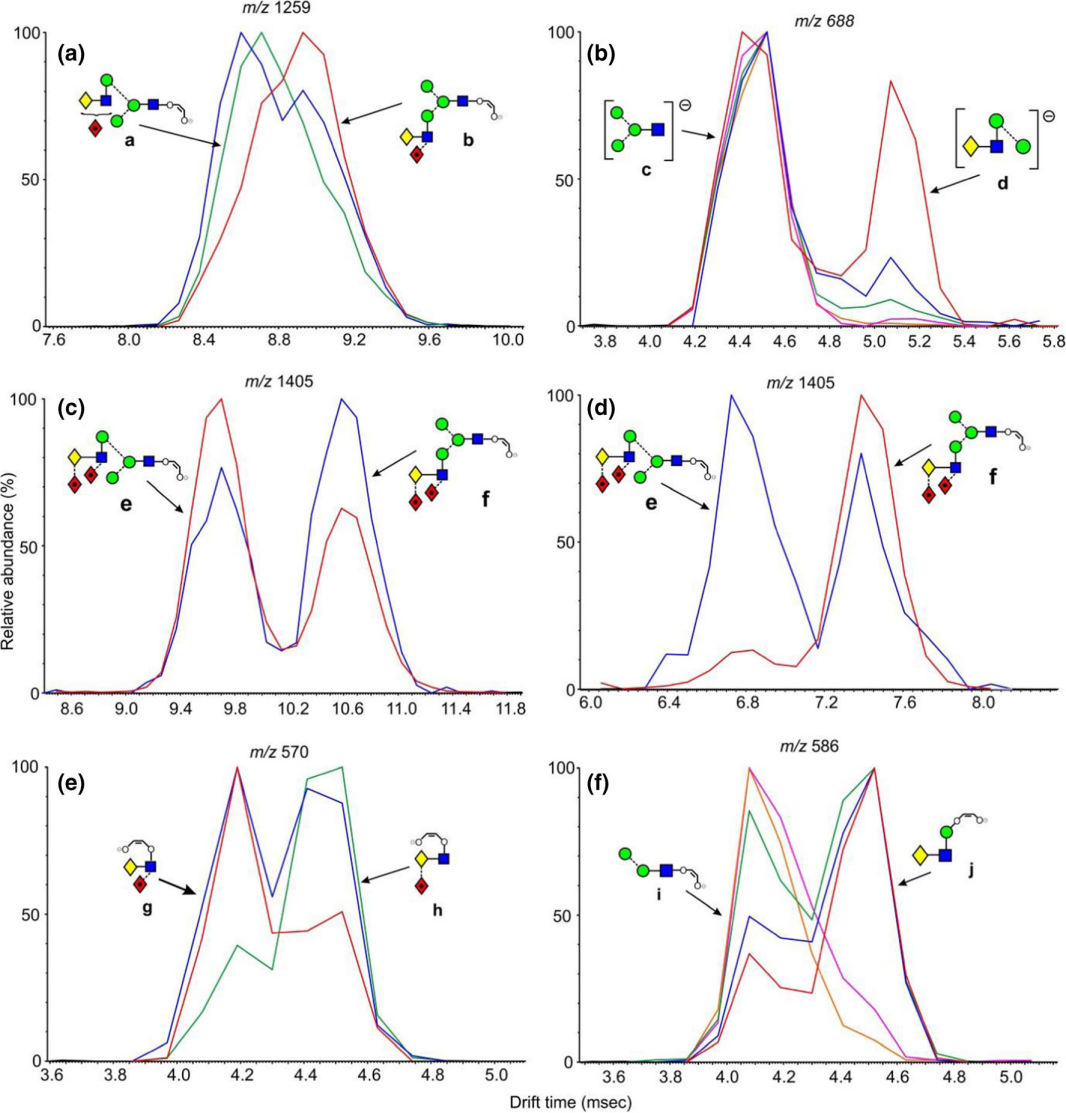
(**a**) Extracted fragment ATD of the ion at *m*/*z* 1259 from fucosylated biantennary glycans. Red trace, glycan **14**; green trace, glycan **21**; blue trace; glycans **17**–**19**. (**b**) Extracted fragment ATD of the ion at *m*/*z* 688 from the fucosylated biantennary glycans. Mono-fucosylated, red trace, di-fucosylated, blue trace, tri-fucosylated, green trace, tetra-fucosylated, pink trace, penta-fucosylated, orange trace. (**c**) Extracted fragment ATD of the ion at *m*/*z* 1405 from the tetra-fucosylated glycan (**19**–**21**, blue trace) and penta-fucosylated glycan (**22**, red trace). (**d**) Extracted fragment ATD of the ion at *m*/*z* 1405 as the ^2,4^A_6_ ion of the hybrid glycan Gal_1_Man_3_GlcNAc_3_Fuc_3_ (**12**, red trace) and the pair of ions from the tetra-fucosylated triantennary glycan Gal_3_Man_3_GlcNAc_5_Fuc_3_ (**19**–**21**, blue trace). The wave velocity on the drift cell (650 m/s) was higher than that used for (**a**). Although this shortened the drift time, it did not significantly affect separation of the doublet. (**e**) Extracted fragment ATD of the ion at *m*/*z* 570. Red trace from glycan **14**; green trace from glycans **19** and **20**, blue trace from glycans **17** and **18**. (**f**) Extracted fragment ATD of the ion at 586 from the fucosylated biantennary glycans: mono-fucosylated, red trace; di-fucosylated, blue trace; tri-fucosylated, green trace; tetra-fucosylated, pink trace; penta-fucosylated, orange trace

As in the spectrum of the hybrid glycans, the di-fucosylated glycan (Fig. 2b) contained prominent ^2,4^A_6_ and B_5_ ions at *m*/*z* 1113 and 1053, the ^2,4^A_6_/Y_4_ and B_5_/Y_4_ ions at *m*/*z* 1113.4 and 1053.4, together with ^1,3^A_3_ ions at *m*/*z* 424 and 570. D and D-18 ions were prominent at *m*/*z* 688 and 670 respectively with minor ions at *m*/*z* 834 and 816, reflecting the virtual absence of fucosylation on the 6-antenna. These ions were consistent with the presence of a core fucose and a fucose substituted on the 3-position of the GlcNAc of the 3-antenna (**14**), as found earlier [43]. A very minor C_2_ fragment at *m*/*z* 325 (Fig. 2b) showed the existence of an additional isomer substituted with fucose on a galactose residue. The earlier work had shown the presence of two such isomers (**15** and **16**). The occurrence of D and D-18 fragments at *m*/*z* 834 and 816 respectively was consistent with the occurrence of isomer **16** but the possible presence of **15** was not determined from the transfer fragmentation spectrum. Consequently, the trap fragmentation spectra were investigated to see if isomer separation could be detected. The *m*/*z* 1259 ion was the only appropriate fragment that showed isomer separation (Fig. 3a). This ion is a fragment of the ^2,4^A_6_ ion that has lost one Gal-GlcNAc group with its attached fucose residues and has the composition (Fuc)Gal-GlcNAc-Man-(Man)Man-GlcNAc-O-CH=CH-O^−^. The di-fucosylated glycan (**14**) produced predominantly a single extracted fragment arrival time distribution (ATD) peak (ion **b**, red trace, Fig. 3a) at this mass, consistent with the presence of glycan **14**. The broad peak from the tetra-fucosylated glycan (**21**, green trace) also appeared to include this structure, as expected, but the main peak had a shorter drift time, probably corresponding to ion **a**. Two peaks (blue trace) were observed from the tri-fucosylated glycans **17** and **18**. One appeared to correspond to ion **b** but, because it would appear from the discussion below on *m*/*z* 570, that all the isomers of this glycan had not been identified in the earlier work, it was not possible to assign structures to the remaining peak and, consequently, differentiation of isomers **15** and **16** by ion mobility was not successful.

The spectrum of the tri-fucosylated glycan from the IgA sample (Fig. 2c) appeared to be of a single compound with fucose substituted on the core and on each of the GlcNAc residues of the antennae (**17**, abundant ^2,4^A_6_/Y_4_ and B_5_/Y_4_ ions at *m*/*z* 1259.4 and 1199.4 respectively, no C_2_ ion at *m*/*z* 325). The spectrum of the tri-fucosylated glycan from the parotid glands, on the other hand (Fig. 2d) was of a mixture of several isomers (**17** and **18** as determined earlier [43]) and at least one unidentified glycan with an antenna containing a single fucosylated galactose residue. As with the hybrid glycans, the core structure with its attached fucose residue was revealed by the masses of the ^2,4^A_R_, B_R-1_ and ^2,4^A_R-1_ fragments. Substitution of a single fucose on the GlcNAc residue of the 3-antenna promoted additional loss of Gal-(Fuc_1_)GlcNAc in a Y_4_ cleavage from the ^2,4^A_6_, B_5_, and ^2,4^A_5_ ions to give *m*/*z* 1259, 1199, and 1056 respectively, together with D and D-18 ions at *m*/*z* 834 and 816 respectively, as above, was consistent with the presence of isomer **17**. Additional losses of the Y_4α_ fragment from the 6-antenna gave the ions at *m*/*z* 748, 888, and 545 (Fig. 2d). In parallel with the formation of the ions at *m*/*z* 1259, 1199, and 1056 were corresponding ions that had lost two fucose residues (*m*/*z* 1113.4, 1053.4, and 910.3) indicating the presence of isomer **18**. The ^1,3^A_4α_ ion at *m*/*z* 716 confirmed the presence of two fucose residues attached to an antenna. A C_2_ ion was present at *m*/*z* 325 showing the occurrence of fucose substitution on the galactose residues. D and D-18 ions, diagnostic for the composition of the 6-antenna, were present at *m*/*z* 688/670 reflecting the lack of a fucose residue in the 6-antenna as would be expected for isomer **18**.

CID spectra of the more highly fucose-substituted biantennary glycans (Fig. 2e, f) showed the same general features as those discussed above. The tetra-fucosylated glycan was found earlier to consist of a mixture of the three isomers **19**, **20**, and **21**. The D and D-18 ions at *m*/*z* 980/972 and at *m*/*z* 834/816 showed the presence of isomers with two and one fucose respectively in the 6-antenna and this was corroborated by two sets of Y_4_ losses from the ^2,4^A_6_, B_5_, and ^2,4^A_5_ ions (*m*/*z* 1405.5/1345.5/1202.4 and 1259.4/1199.4/1056.4 respectively. Prominent C_2_ ions at *m*/*z* 325 confirmed fucose substitution on galactose consistent with the occurrence of isomers **20** and **21**. The presence of isomer **20** was confirmed by the presence of the ions at *m*/*z* 427 and 409 in the CID spectrum. These ions arise from an ^0,2^A_2_ cleavage of the antenna GlcNAc residue and are only seen in glycans with a Fuc-α1→2-Gal-β1→4GlcNAc moiety, but it was not possible to say which isomer this ion came from. Consequently, the presence of isomer **20** could not be confirmed. Finally, Fig. 2f shows the spectrum of the penta-fucosylated glycan (**22**) with the major diagnostic ions labeled. The absence of the C_1_ ion at *m*/*z* 179 and the presence of *m*/*z* 325 are consistent with fucose substitution on both galactose residues.

#### Ion Mobility

**Figure 4:**
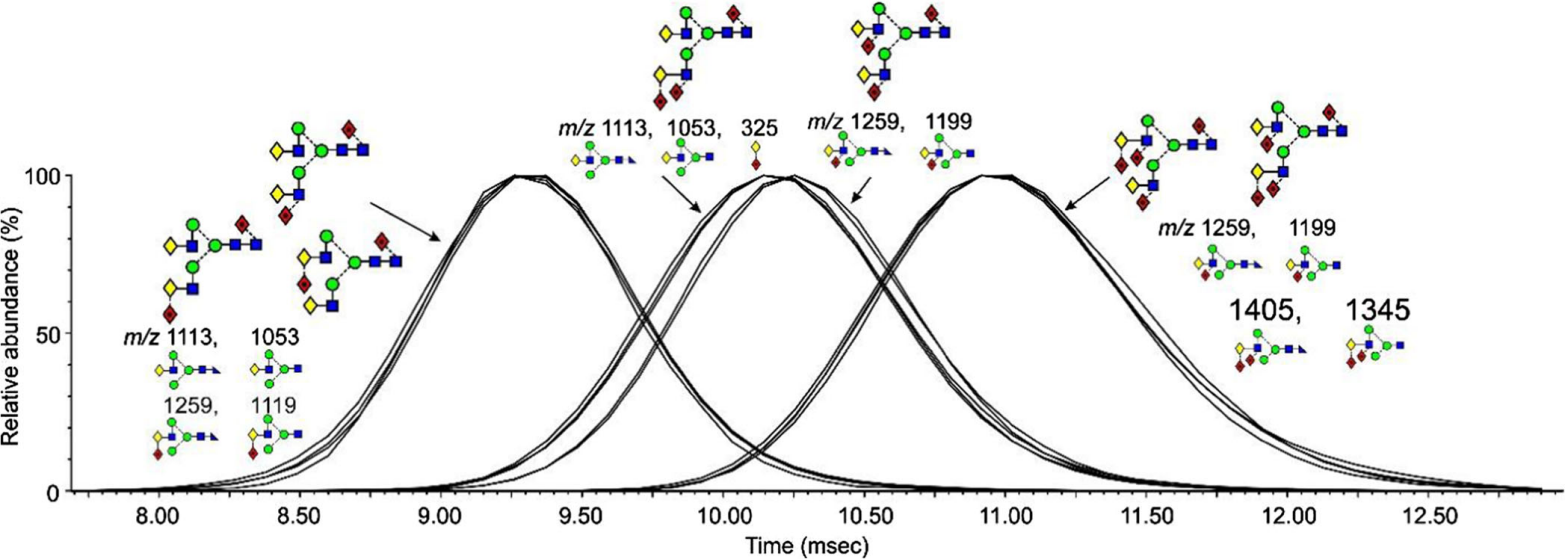
Extracted fragment ATDs of fragment ions generated in the transfer fragmentation spectrum reflecting partial separation of the intact tri-fucosylated biantennary isomers (**17** and **18**). Structures of the ions are shown below their masses. The peaks on each side of the separated peaks from the tri-fucosylated glycans are from corresponding fragments from the di- and tetra-fucosylated glycans where no isomeric separation could be detected

The capability of ion mobility to separate isomers of the bi-, tri-, and tetra-fucosylated biantennary glycans was examined. Two isomers (**17** and **18**) of the tri-fucosylated biantennary glycans (*m*/*z* 2175.7) were present in the parotid sample. Extracted fragment ATDs of the D, D-18, and ^0,4^A_4_ ions (*m*/*z* 688, 670, 616 and 834, 816, 762 respectively) for glycans **17** and **18** showed a small difference in drift time for the D and D-18 ions (masses as above) suggesting small differences in cross section and this was confirmed by extracted fragment ATDs for the pairs of ions at *m*/*z* 1113.4, 1053.4 and 1259.5, 1199.5 which are formed by loss of the 3-antenna from the ^2,4^A_7_ (*m*/*z* 1770.6) and ^2,4^A_6_ (*m*/*z* 1710.6) respectively (Fig. 4). The extracted fragment ATD of *m*/*z* 325 (C_1_ fragment from the di-fucosylated 3-antenna) showed coincidence with the extracted fragment ATDs of the ions (*m*/*z* 1113.5 and 1199.4) that had lost the di-fucosylated 3-antenna. However, because of the very small differences in the measured cross sections, these differences would not be a reliable indicator of structure. No corresponding separations were observed for the isomers (**19**, **20** and **21**) of the tetra-fucosylated glycans (*m*/*z* 2321.8, Fig. 4) or of the bi-fucosylated glycans.

#### Trap Fragmentation

**Figure 5:**
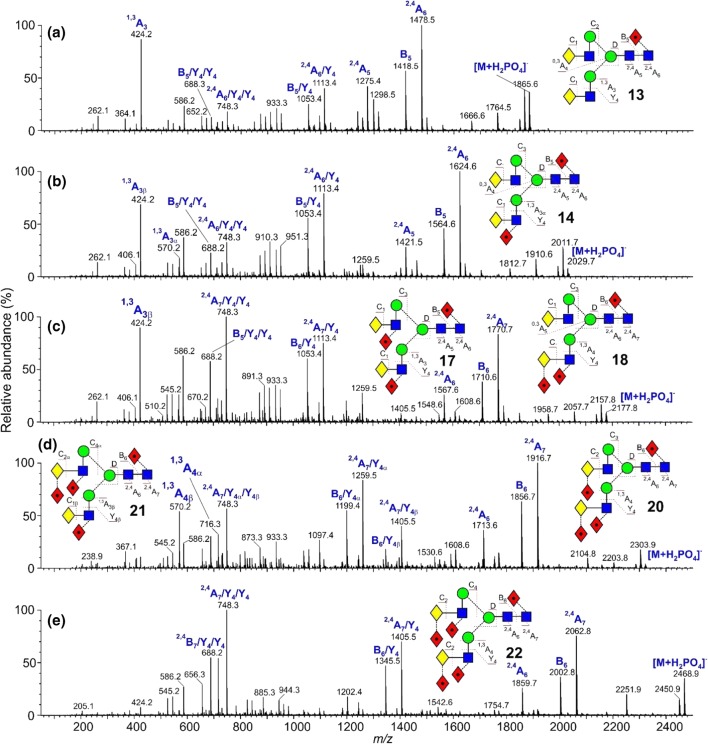
(**a**–**e**) Negative ion CID spectra (trap region) of fucosylated biantennary complex glycans from parotid gland extracts. Symbols and linkages for the structural diagrams are as defined in the footnote of Table 1

The trap CID spectra of the five biantennary glycans from parotid glands are shown in Fig. 5. Significant differences were noted between these spectra and the transfer fragmentation spectra. Although the ions in the upper portion of the spectra were the same in both types of spectra, those ions at low mass were different. In particular, the C_1_ (C_2_ fragment when fucose was attached to the galactose residue) ions were absent and likely not transmitted through the IM cell. The C_2_ fragment from Fuc-Gal-containing glycans (*m*/*z* 325), in particular, is an important ion for locating the fucose to a particular position of the antennae.

The trap fragmentation spectra contained a greater proportion of Y-type secondary and tertiary cleavages than the transfer spectra as is particularly noticeable in the spectrum of the penta-fucosylated glycan (Fig. 5e). Thus, the ^2,4^A_7_, B_6_, and ^2,4^A_6_ (*m*/*z* 2062, 2002, and 1859 respectively) fragmented by additional Y_4_-type cleavages to give the ions at *m*/*z* 1405, 1345, and 1202 respectively and by two additional Y_4_-type cleavages to give *m*/*z* 748, 688, and 545. Consistent with the loss of all antenna-attached fucose residues, these latter ions were present in all five spectra. Unfortunately, the ion at *m*/*z* 688 was isobaric with the D ion containing no fucose residues which, combined with the fact that the spectra contained a group of several ions with comparable relative abundance, made the identification of the 6-antenna-specific D and D-18 ions difficult. These ions were prominent in the transfer spectra. Initial work with complex glycans (unpublished results) showed that D-type ions at *m*/*z* 688 exhibited a unique cross section and when the profile of *m*/*z* 688 was examined. The extracted fragment ATD of *m*/*z* 688 from the fucosylated biantennary glycans (Fig. 3b) clearly identified this ion (red trace, ion **d**). Its relative abundance decreased progressively with increasing numbers of fucose residues such that the glycans with four and five residues did not contain a D ion at *m*/*z* 688. The ^1,3^A ion at *m*/*z* 424 was very prominent in spectra lacking a fucose-substituted antenna but the fucose-substituted analogues (*m*/*z* 570 and 716) were less prominent than in the transfer spectra. Thus, the transfer CID spectra were much more useful in the identification of these glycans than the trap CID spectra.

Some peak broadening was observed in the ATDs of a few of the fragments recorded in the trap region but three other ions produced significant doublets. Thus, the ^2,4^A_7_/Y_4_ ions at *m*/*z* 1405 from the tetra-fucosylated (**19**–**21)** and penta-fucosylated glycans (**22**) showed well-separated doublets (Fig. 3c) produced from loss of either antenna from the ^2,4^A_7_ ions. The extracted fragment ATD from the hybrid glycan Gal_1_Man_3_GlcNAc_3_Fuc_3_ (**4**) showed the same drift time as the peak with the larger drift time from the biantennary glycans consistent with the structure of ion **f** (red trace, Fig. 3d). The other ion, therefore, has structure **e**.

The cross-ring ion at *m*/*z* 570 containing galactose, GlcNAc, and fucose could have two structures depending on whether the fucose was located on the galactose or GlcNAc residue (Gal-(Fuc)GlcNAc-O-CH=CH-O^−^ and (Fuc)Gal-GlcNAc-O-CH=CH-O^-)^. The *m*/*z* 570 trap fragmentation ATD showed two peaks consistent with these structures. A third structure could be (Fuc)GlcNAc-Man-CH=CH-O^−^ but this is unlikely because no evidence has been found for the corresponding ion without fucose (*m*/*z* 424). The transfer fragmentation spectrum of the di-fucosylated biantennary glycan (**14**, Fig. 2b) contained only a trace of the ion at *m*/*z* 325 (Fuc-Gal) showing that most of these glycans contained fucose attached to the GlcNAc residue. Therefore, the extracted ATD peak for *m*/*z* 570 (one fucose residue) from the di-fucosylated glycans (**14**, ion **g**) should correspond to the larger of the peaks, i.e., that with the shortest drift time (red trace, Fig. 3e). The paper by Guile et al. reported that the major tetra-fucosylated glycan contains two fucose residues on the 3-antenna and one on the galactose of the 6-antenna (glycans **19** and **20**). Its CID spectrum (Fig. 2e) contained prominent fragments at *m*/*z* 427 and 409 (^0,2^A_4_ and ^0,2^A_4_–H_2_O respectively) which recent work (unpublished) has shown to be diagnostic for the presence of fucose linked to galactose. Thus, the isomer with the single fucose in the 6-antenna is compound **20** consistent with the second of the ATD peaks (green trace) from *m*/*z* 570. ATDs of this fragment, therefore, are diagnostic for fucose linked either to GlcNAc (first ATD peak) or to galactose (second peak). The extracted fragment ATD of *m*/*z* 570 from the tri-fucosylated glycans (**17**, **18**) produced two peaks (blue trace, Fig. 3e) whose drift times corresponded to these two structures. Furthermore, Guile et al. only reported tri-fucosylated biantennary glycans with singly fucosylated antennae where the fucose was located on the GlcNAc residue. However, the transfer fragmentation spectrum showed a prominent Fuc-Gal ion at *m*/*z* 325 which, together with the ion mobility data from *m*/*z* 570 (two peaks), shows the presence of at least one additional isomer that was missed in the earlier work. Although the presence of the fragments at *m*/*z* 427 and 409 indicate the occurrence of compounds with fucose attached to galactose, it is difficult to determine the relative contribution of this moiety in mixed spectra. The relative abundance of the extracted fragment ATD peaks goes some way towards providing this information.

**Scheme 1:**
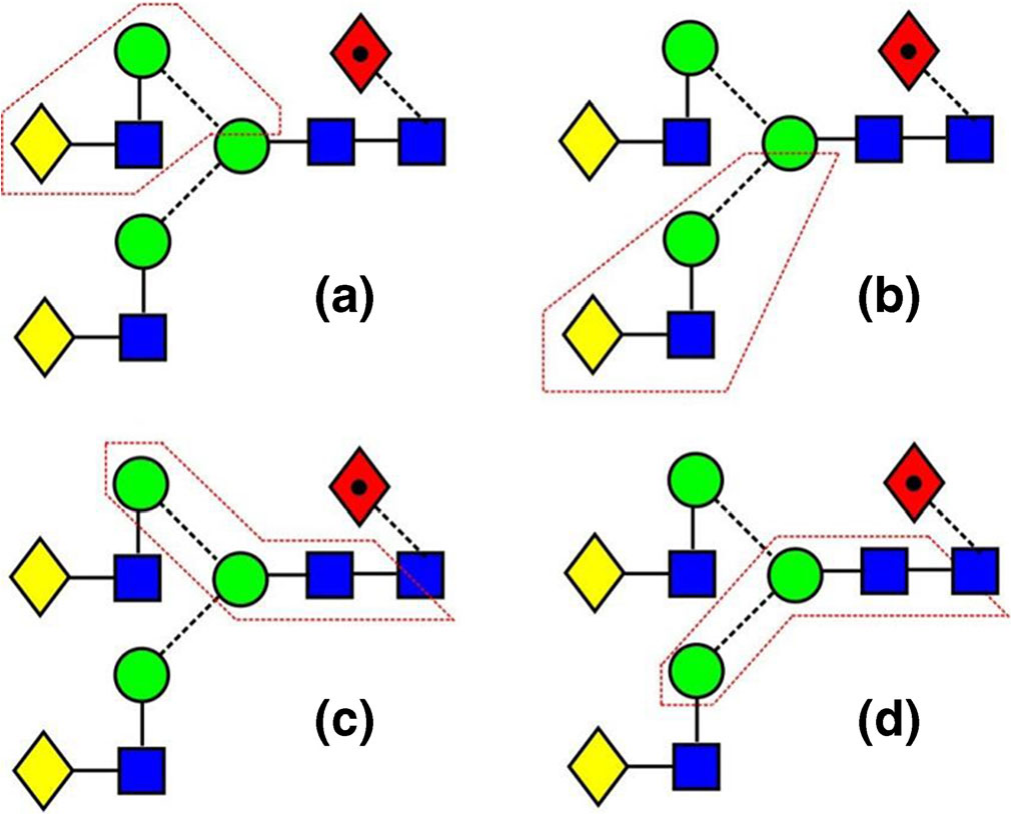
Biantennary glycans showing possible cleavages (surrounded by red boxes) giving rise to the fragment ion at *m*/*z* 586

A third ion that showed separation of fragment structures was *m*/*z* 586. This ion has the composition Hex_2_GlcNAc_1_-O-CH=CH_2_-O^−^ and can arise from several regions of the biantennary glycans as shown in Scheme 1. Figure 3f shows the fragment ion ATDs of this ion recorded from the five biantennary parotid glycans. The profile of the doublet changes according to the number of fucose residues on the antennae. Substitution of one fucose on the 3-antenna does not change the profile indicating that pathway (**b**) is insignificant. In the profile from the penta-fucosylated glycan (**22**) the second peak is absent consistent with its being formed by pathway (**a**). Consequently, the first peak is probably formed via pathway (**c**) or (**d**).

The use of trap fragmentation combined with ion mobility to determine the location of the fucose residues has been discussed in an earlier paper [71].

### Triantennary Glycans

**Figure 6:**
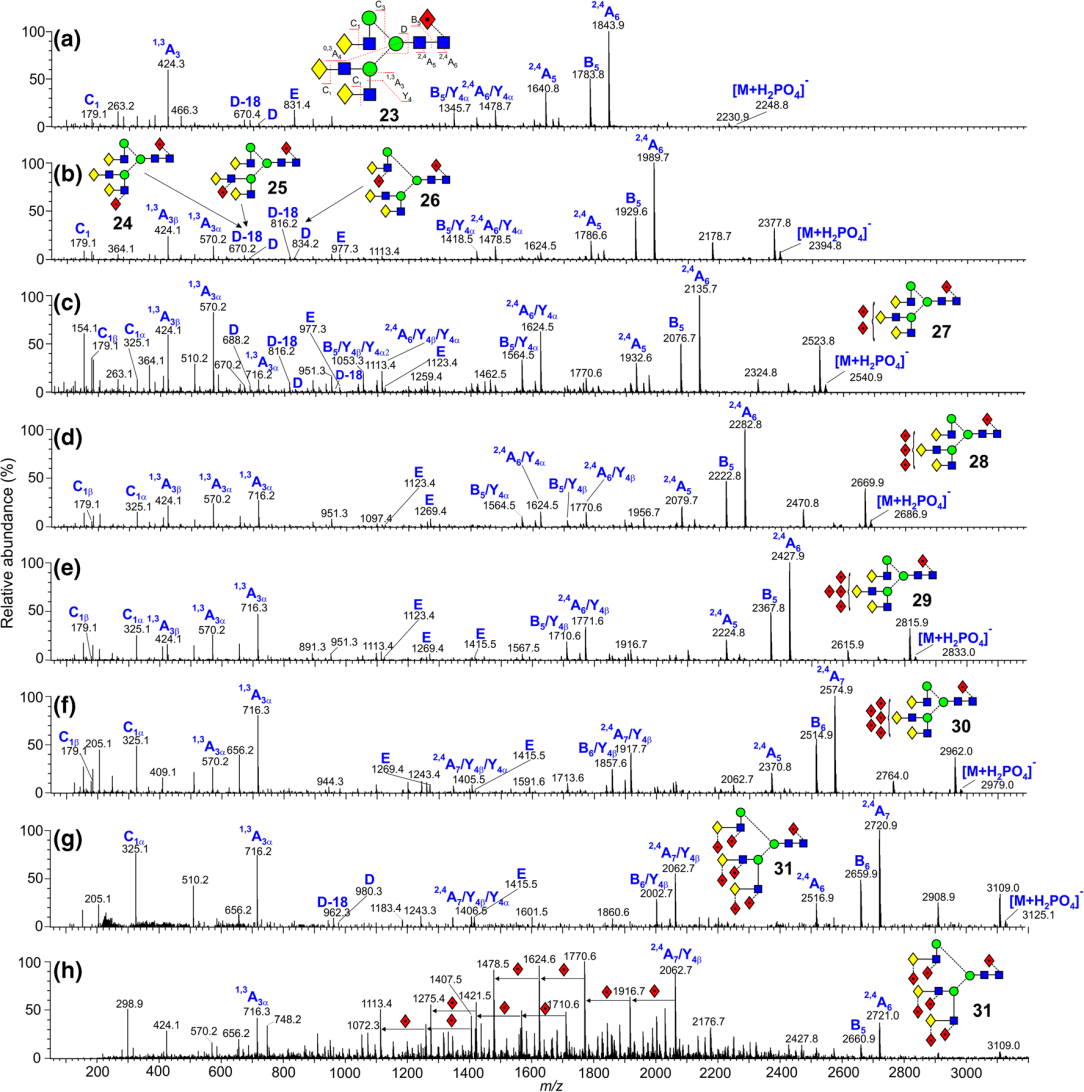
(**a**–**g**) Negative ion CID spectra (transfer region) of fucosylated triantennary complex glycans (**23**–**31**) from human parotid gland extracts. (**h**) Trap fragmentation spectrum of the hepta-fucosylated triantennary glycan **31**. Symbols and linkages for the structural diagrams are as defined in the footnote to Table 1. Nomenclature of the ions is given in (**a**)

Again, all glycans contained fucose at the 6-position of the reducing-terminal GlcNAc residue as shown by the masses of the ^2,4^A_R_, B_R-1_, and ^2,4^A_R-1_ ions in the transfer CID spectra (Fig. 6). The spectrum of the mono-fucosylated glycan (**23)** contained a prominent fragment at *m*/*z* 831 (labeled as ion E) and D and D-18 ions at *m*/*z* 688 and 670 respectively showing that the glycan contained a branched 3-antenna [72]. This method for determining the branching pattern of the triantennary glycans is much more rapid than the methylation linkage method used in the original work that required three stages of derivatization and analysis by GC/MS. The results obtained by the negative ion method were fully consistent with those obtained earlier. Shifts in the masses of the D, D-18 ions and *m*/*z* 831 easily located the positions of the fucose residues, as described above.

The spectrum of the di-fucosylated glycan, for which three isomers (**24**, **25**, and **26**) had been found earlier, contained both ions *m*/*z* 424 and its fucosylated analogue *m*/*z* 570 showing that the second fucose was on an antenna. The ion at *m*/*z* 831 had mainly shifted to *m*/*z* 977 showing that the fucose was substituted mainly on the 3-antenna and that only a trace of the C_2_ ion at *m*/*z* 325 (Fuc-Gal) showed that it was not attached to the galactose residue, again consistent with the earlier work. The extracted fragment ATD showed predominantly ion **g** (Fig. 3e, red trace) with a minor amount of ion **h**, in agreement with this finding. The D and D-18 ions at *m*/*z* 688 and 670 respectively (no fucose) were consistent with substitution in the 3-antenna but minor ions 146 mass units higher showed a small amount of fucose substitution in the 6-antenna. Relative amounts of these isomers were not reported earlier but the current results show a preponderance of fucose substitution on the 3-antenna. No extracted fragment ATDs were found to enable the branch of substitution to be established.

The spectrum of the tri-fucosylated glycan (**27**) showed it to be a mixture of several isomers. The earlier work reported only two isomers containing a core fucose and fucose residues attached to the GlcNAc residues of unspecified antennae. No isomers with fucose attached to galactose were detected. Negative ion CID, however, showed the presence of additional isomers, some of which were substituted on this residue (presence of the C_2_ ion at *m*/*z* 325). E-type ions at *m*/*z* 977 and 1123 showed the presence of isomers with one and two fucose residues respectively on the 3-antenna and ions at *m*/*z* 424, 570, and 716 indicated the existence of Gal-GlcNAc-moieties containing antennae with zero, one, and two fucose residues respectively. Of these ions, *m*/*z* 570 was more abundant than the other two showing that isomers with mono-substitution of fucose residues on individual antennae were the most abundant. Absence of the ion at *m*/*z* 831 showed that at least one fucose was in the 3-antenna and this was confirmed by the presence of its analogue at *m*/*z* 977 (one fucose) and 1123 (two fucose residues). Thus, the isomers appeared to have either one or two fucose residues on the 3-antenna (either on the same or different branches) or to have one fucose residue on each antenna. However, the location of the fucose to galactose or GlcNAc within each isomer was not determined. From the discussion above, the drift times of the ion at *m*/*z* 1405 can be used to determine the presence of difucosylation on the 3- or 6-antennae. The relative abundance of *m*/*z* 1405 in the trap fragmentation spectrum of the tri-fucosylated triantennary glycan was low but its drift time plot produced the same two peaks as shown in Fig. 3c, d consistent with the presence of isomers containing difucosylation in both the 6- and 3-antennae, probably the 3-branch, and consistent with the CID data. However, without authentic standards, this information was not sufficient to determine if difucosylation occurred on the 4-branch of the 3-antenna.

The spectra of the more highly substituted triantennary glycans (**28**–**31**) showed them to be mixtures of isomers with a complex distribution of fucose residues among the antennae. All contained the ions at *m*/*z* 716 and 656 ((Fuc)Gal-(Fuc)GlcNAc with and without –O-CH=CH-O^−^) showing the presence of two fucose residues on at least one antenna. D and E ions in the spectrum of the glycan with seven fucose residues (*m*/*z* 980 and 1415 respectively) confirmed the presence of two fucose residues in the 6-antenna and four in the 3-antenna (glycan **31**). The absence of ion **j** in the trap fragmentation profile of *m*/*z* 586 (Fig. 3f) from the hexa- and hepta-fucosylated glycans **30** and **31** was consistent with all antennae containing at least one fucose. Its presence in all other glycans showed at least one unfucosylated antenna.

#### Trap Fragmentation

As with the biantennary glycans, the trap fragmentation spectra were less informative than the transfer spectra for determination of structure because of the greater proportion of secondary and tertiary fragments at the expense of ions such as the D and E fragments. Furthermore, additional ions produced by fucose neutral losses were observed in the spectra of the more heavily fucosylated glycans such as in the spectrum of the penta-fucosylated triantennary glycan (**31**) shown in Fig. 6h.

### Bisected Biantennary Glycans

**Figure 7:**
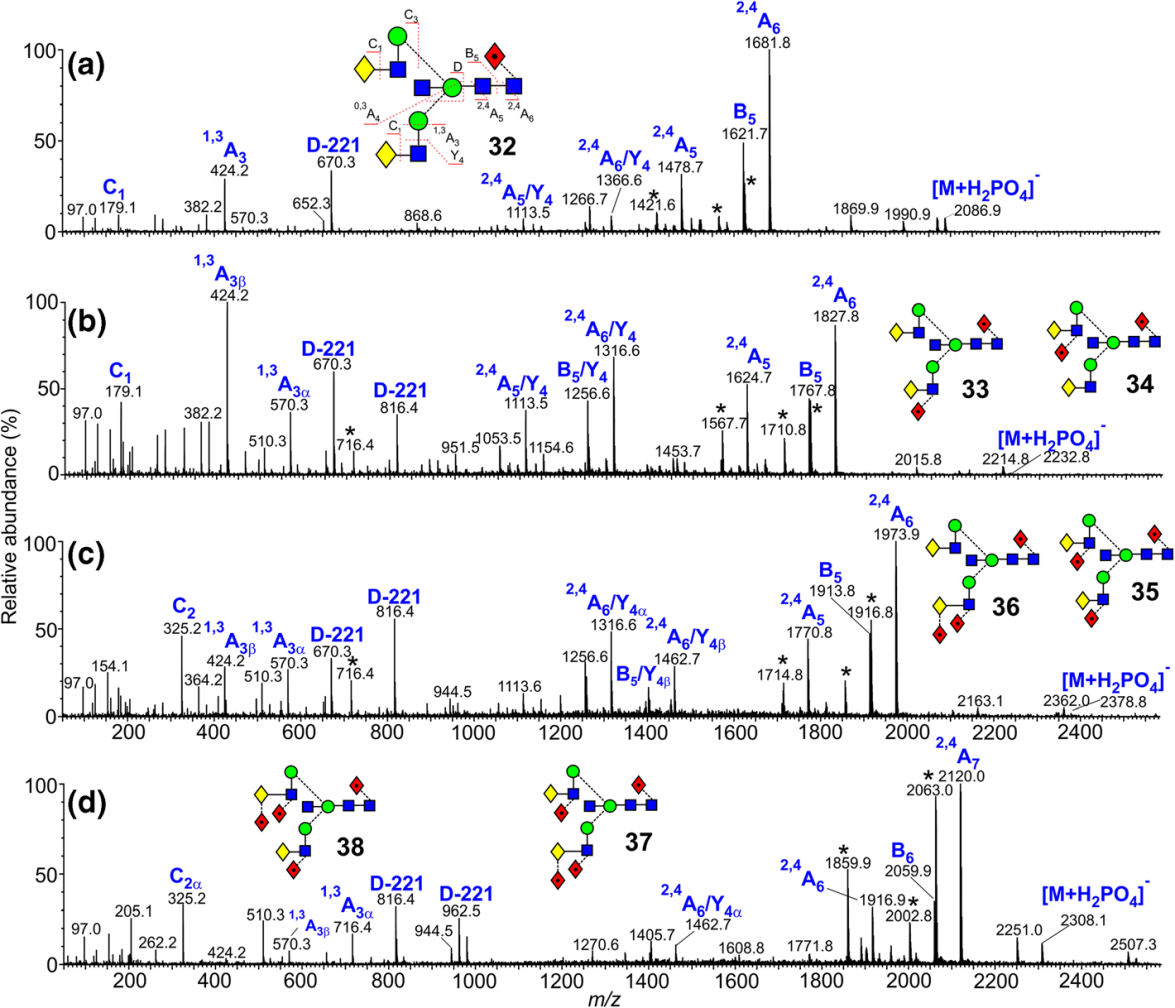
(**a**–**d**) Negative ion CID spectra (transfer region) of bisected biantennary complex glycans (**32**–**38**) from human parotid gland extracts. Symbols and linkages for the structural diagrams are as defined in the footnote of Table 1. Nomenclature of the ions is shown in (**a**). Ions marked with an asterisk are from isobaric biantennary glycans containing an additional *N*-acetylamino group, produced as a by-product of the hydrazinolysis-reacetylation release method

Spectra of the bisected biantennary glycans with one to four fucose residues are shown in Fig. 7. Unfortunately, the spectra were contaminated with what appeared to be artifacts of the hydrazinolysis release method produced by addition of an additional acetylamino group to the reducing terminus of the un-bisected biantennary glycans [73]. Major ions from this reaction are indicated with an asterisk in the figure. Ions from these glycans at the high-mass end of the spectrum correspond to ^2,4^A_5_, B_4_, and ^2,5^A_4_ fragments, formation of the corresponding ^2,4^A_6_ ions having been prevented because of the open-ring nature of the reducing-terminal GlcNAc residue. The glycans did not show any significant differences in drift time and could not, thus, be separated by ion mobility. However, structural information on the bisected glycans could still be obtained from the negative ion CID spectra because of the production of unique ions specific to the presence of the bisecting GlcNAc. These spectra provided additional information on the structure of these glycans than was obtained in the original work.

Instead of the production of the D and D-18 pair of ions from the 6-antenna, bisected glycans produce a prominent ion resulting from loss of the bisecting GlcNAc residue from the D ion (termed the D-221 ion) together with a second ion arising from an additional loss of water [16, 17]. The D ion is missing. In the spectrum of the mono-fucosylated glycan (**32**, Fig. 7a), this pair of ions can be seen at *m*/*z* 670 (labeled D-221) and 652 respectively. Movement of these ions to *m*/*z* 816 and 962 indicates the addition of one and two fucose residues respectively. Thus, in the spectrum of the bi-fucosylated glycan (**33**, **34**), the presence of these ions at *m*/*z* 670 and 816 is consistent with the substitution of a single fucose residue on either antenna. The extracted fragment ATD of ion **b** (*m*/*z* 1259) showed that isomer **33** predominated. The low relative abundance of the C_2_ fragment at *m*/*z* 325 is consistent with the earlier report that the fucose residues are mainly located on GlcNAc residues. Possible ATD fragments indicating the antenna to which the fucose was attached were not identified.

Although the earlier work reported the presence of additional bisected glycans with three and four GlcNAc residues, the distribution of the fucose residues was not established other than that they appeared to be mainly attached to GlcNAc residues. Figure 7c shows the CID spectrum of the tri-fucosylated biantennary glycans (**35**, **36**). It contained a very prominent D-221 ion at *m*/*z* 816 (one fucose residue in the 6-antenna) and a less abundant analogue at *m*/*z* 670 which, together with the ^2,4^A_4_ ion at *m*/*z* 716 showed di-fucose substitution on the 3-antenna. The isomers, therefore, appeared to be **35** and **36** (Table 1). Although the presence of the contaminating compound distorted the appearance of the spectrum of the tetra-fucosylated glycan (Fig. 7d), the two D-221 ions at *m*/*z* 816 and 962 (not present from the contaminant) showed the presence of one and two fucose residues respectively in the 6-antenna, consistent with the structures **37** and **38**.

## Conclusions

The work reported here demonstrates the ability of negative ion CID coupled with ion mobility to provide significant structural information on fucosylated *N*-glycans. Specifically, the spectra present information on which antenna and residues bear the fucose substituents and how many of each are on which specific antenna. Such information is principally available from exoglycosidase data but only after several rounds of digestion. The fragmentation information, however, does not provide direct information on the linkage (1→2 or 1→3) of the fucose residues and, therefore, this information must still be obtained by traditional techniques. The spectra also provide direct information on the topology of the glycans (e.g., bisected and type of triantennary glycan).

Transfer fragmentation data provided the most useful structural information because the corresponding trap fragmentation spectra tended to lack some of the low mass diagnostic ions and also to contain secondary and tertiary fragments. This observation complicated the identification of specific ions such as the D-fragment that are informative for the composition of the 6-antenna. However, this information could often be recovered by examination of the extracted fragment ATDs where, for example, the ion at *m*/*z* 688 (Gal-GlcNAc-Man-Man^−^) gave a unique drift time. Extracted fragment ATDs of several other fragments gave different drift times depending on their composition. Predominant among these was *m*/*z* 1405 that differentiated antennae contained two fucose residues.

In conclusion, the work demonstrates the ability of negative ion CID coupled with ion mobility mass spectrometry to provide significant structural information rapidly from native glycans and illustrates how ion mobility of fragment ions adds a further technique that is proving useful for structural determination of these complex molecules.

## Abbreviations

ATD: Arrival time distribution
CCSs: Collisional cross sections
CID: Collision-induced dissociation
ESI: Electrospray
Fuc: Fucose
Gal: Galactose
GC/MS: Combined gas chromatography/mass spectrometry
Glc: Glucose
GlcNAc: *N*-Acetylglucosamine
HDMS: High-definition mass spectrometer
IgA: Immunoglobulin A
IMS: Ion mobility
Man: Mannose
PNGase F: Protein *N*-glycosidase F
Q: Quadrupole
TOF: Time-of-flight
TWIMS: Traveling wave ion mobility
